# Disposable Sensor Chips with Molecularly Imprinted Carbon Paste Electrodes for Monitoring Anti-Epileptic Drugs

**DOI:** 10.3390/s23063271

**Published:** 2023-03-20

**Authors:** Aaryashree, Ashish Kumar Choudhary, Yasuo Yoshimi

**Affiliations:** 1 Innovative Global Program, Shibaura Institute of Technology, Toyosu, Koto-City, Tokyo 135-8548, Japan; aarya@sic.shibaura-it.ac.jp; 2 Department Applied Chemistry, Shibaura Institute of Technology, Toyosu, Koto-City, Tokyo 135-8548, Japan

**Keywords:** phenobarbital, carbamazepine, levetiracetam, molecular imprinting, carbon paste, disposable sensor

## Abstract

*Epilepsy* is a neurological disorder that affects millions of people worldwide. Anti-epileptic drugs (AEDs) are critical for their management. However, the therapeutic window is narrow, and traditional laboratory-based therapeutic drug monitoring (TDM) methods can be time consuming and unsuitable for point-of-care testing. To address this issue, we developed a disposable sensor chip based on molecularly imprinted polymer-modified carbon paste electrodes (MIP-CPs) for the TDM of AEDs such as phenobarbital (PB), carbamazepine (CBZ), and levetiracetam (LEV). In this work, functional monomers (methacrylic acid) and crosslinking monomers (methylene bisacrylamide and ethylene glycol dimethacrylate) were copolymerized in the presence of the AED template and grafted on the graphite particles by simple radical photopolymerization. The grafted particles were mixed with silicon oil, dissolving ferrocene as a redox marker to make the MIP-carbon paste (CP). Disposable sensor chips were fabricated by packing the MIP-CP into the base made of poly (ethylene glycol terephthalate) (PET) film. The sensor’s sensitivity was determined using differential pulse voltammetry (DPV), carried out on a single sensor chip for each operation. Linearity was obtained from 0–60 μg/mL in PB and LEV and 0–12 μg/mL in CBZ, covering their respective therapeutic range. The time taken for each measurement was around 2 min. The experiment using whole bovine blood and bovine plasma indicated that the existence of species that interfered had a negligible effect on the test’s sensitivity. This disposable MIP sensor provides a promising approach for point-of-care testing and facilitating the management of epilepsy. Compared with existing tests, this sensor offers a faster and more accurate way to monitor AEDs, which is crucial for optimizing therapy and improving patient outcomes. Overall, the proposed disposable sensor chip based on MIP-CPs represents a significant advancement in AED monitoring, with the potential for rapid, accurate, and convenient point-of-care testing.

## 1. Introduction

Anti-epileptic drugs (AEDs) are the primary method of therapy for epilepsy, a persistent neurological brain illness affecting about 50 million people worldwide [1,2,3]. The proper therapeutic management of AEDs requires monitoring their levels in the blood to ensure efficacy and prevent toxic side effects. Currently, the monitoring of AEDs is performed by analyzing blood plasma, serum, or saliva samples in laboratories using various analytical techniques such as liquid chromatography or mass spectrometry [4,5,6,7,8,9,10]. However, these techniques are time consuming, require specialized equipment and trained personnel, and are unsuitable for real-time or in-situ monitoring.

Electrochemical sensors have recently gained attention as a promising alternative for analyzing the AEDs [10,11,12]. Electrochemical approaches have several advantages over traditional analytical methods, including fast analysis, sensitivity, selectivity, low cost, and no sample pre-treatment or separation requirement [13,14]. Due to these benefits, electrochemical analysis methods have become popular in the pharmaceutical analysis market. They have replaced time-consuming chromatographic methods in clinical analysis, quality control, and the routine determination of drugs.

Carbon paste electrodes are widely used in electrochemical analysis because of their high electrical conductivity, low cost, and ease of preparation [15,16,17]. Carbon paste electrodes are a mixture of graphite powder and a conducting binder. The high surface area for electron transfer and porous structure of the carbon paste electrode makes it particularly well-suited for the electrochemical analysis [18,19,20,21,22]. Carbon-based electrodes are modified by adding chemical or biological components to their surfaces to improve electrochemical performance. The modification can be carried out through various techniques such as chemical adsorption, physical deposition, and covalent bonding. The modifications allow for improved selectivity, sensitivity, stability, and reaction kinetics, making them ideal for various applications.

One of the several techniques of carbon surface modification is the molecular imprinting technique. It is a process in which a specific molecule is used as a template to create a molecular imprint in a polymer matrix. The resulting molecularly imprinted polymer (MIP) has the unique ability to selectively recognize and capture the target molecule in a complex mixture, providing a highly selective and sensitive method for its analysis. Because of this, MIPs have demonstrated immense potential in a wide range of applications, including chemical and biological sensing such as nanosensing [23,24], separation [25,26], drug delivery [27,28], and catalysis [29,30]. MIPs have been used in chemical sensing to develop highly selective and sensitive sensors for various analytes, including environmental pollutants, food contaminants, and drugs [31,32]. In addition, MIPs have been utilized in the separation of complex mixtures and in drug delivery systems to control the release of drugs in a targeted and sustained manner. The versatility of MIPs in different applications makes them highly attractive for use in various fields, including healthcare, environmental monitoring and regeneration, and the food industry.

In recent years, there have been several studies on using MIPs for AED detection, with some researchers reporting detection limits in the nanomolar range and being capable of detection in biological fluids. However, despite these promising results, there is still a need for the development of MIP sensors that can provide reliable and accurate detection of AEDs in a wide range of conditions, including those that may affect sensor performance, such as pH, temperature, and interference from other substances. One of the significant limitations of present MIP-based sensors is the lack of robustness and reproducibility of MIP synthesis protocols, which can lead to batch-to-batch variation in sensor performance. Additionally, many MIP-based sensors need more selectivity and sensitivity, mainly when applied to complex matrices such as biological fluids. There is also a need for further validation of MIP-based sensors against established analytical methods and clinical samples to assess their accuracy and reliability in real-world settings. Finally, there need to be more studies that explore the scalability and commercial viability of MIP-based sensors, which is a critical aspect of their widespread adoption in clinical practice.

In this study, we aimed to develop a disposable chip sensor for therapeutic drug monitoring (TDM) of AEDs by utilizing a molecularly imprinted polymer (MIP)-modified carbon paste electrode. The MIP-CP-based sensing material on a disposable chip sensor provides a simple and cost-effective fabrication process for the sensor, making it more accessible for scale-up and mass production. Furthermore, this sensor offers several advantages, such as portability for field-based applications, reduced sample contamination, reagentless sensing, and selective analyte detection in a wide range of biological matrices. Although this work focused on the phenobarbital sensor, we also presented preliminary work on levetiracetam and carbamazepine sensors. 

## 2. Materials and Methods

### 2.1. Materials and Instruments

Methacrylic acid (MAA), *N*,*N*’-methylene bisacrylamide (MBAA), acrylamide (AAm), ethylene glycol dimethacrylate (EDMA), phenobarbital sodium salt (PB), and carbamazepine (CBZ) were purchased from Wako Pure Chemical Industry (Osaka, Japan). Levetiracetam (LEV) and ferrocene were purchased from Tokyo Chemical Industry (Tokyo, Japan). *N*,*N*-dimethylformamide (DMF) was purchased from Kanto Chemical Co., Ltd. (Tokyo, Japan). Bovine blood for testing was bought from the Tokyo Shibaura Zoki Corporation (Tokyo, Japan). The graphite particles (SG-BH8, 8 μm in diameter) were generously donated by Ito Graphite Co., Ltd. (Kuwana, Japan). Silicone oil (300 cs viscosity) was purchased from Shin-Etsu Chemical Co., Ltd. Heat-adhesive polyethylene terephthalate (PET) sheets (Nakabayashi, LPR-A4E2) were purchased from Amazon online shop. Conductive carbon ink (RAFS 090) was obtained from Toyo Ink Co., Ltd., Tokyo, Japan. Silver/Silver Chloride was purchased from ALS Co., Ltd. (Tokyo, Japan). Distilled water was prepared automatically from WG-204 (Yamato Scientific Co., Ltd., Tokyo, Japan).

JSM-6010LV (JEOL) was used for scanning electron microscopy images. Hamamatsu L9588 LightningCure spot-light source Model LC8 was used for the UV source. JET-Circuit (*e*pronics Co., Ltd., Tokyo, Japan) printer was used to print conductive ink (JC-IC3, *e*pronics Co., Ltd.) on the specified PET sheets (JC-PETA410, *e*pronics). Fellowes-Jupiter Plus laminator was used for laminating the sheets. A fabool-CO_2_ laser cutter (smartDIY Co., Ltd., Minami Alps, Japan) was used for cutting the PET sheets. For all the electrochemical analyses, Compactstat. h. potentiostat (Ivium Technologies, Eindhoven, the Netherlands) installed with IviumSoft version 4.1084 [4] was utilized.

### 2.2. Synthesis of Molecularly Imprinted Polymers

**Figure 1:**
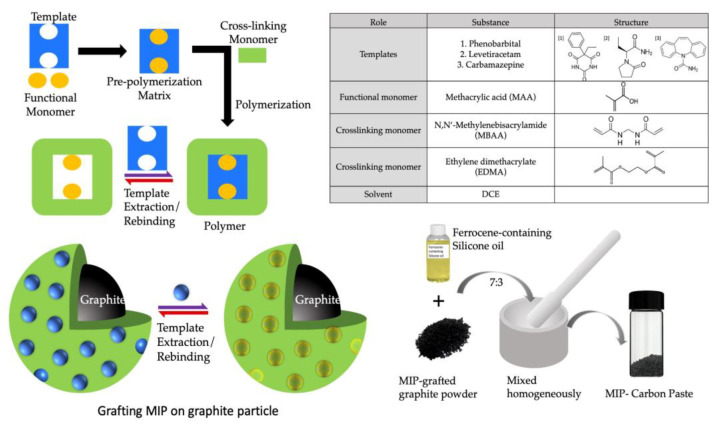
Schematic preparation of MIP by radical photopolymerization and obtaining MIP-CP from MIP powder by homogenous mixing of MIP in ferrocene-containing silicone oil.

To create the initiator graphite (IG), a diethyldithiocarbamate methylene group was added to the surface of the graphite particle via chloromethylation through the procedure described previously [22]. A general radical polymerization procedure was used to create the MIP for all the templates/analyte drugs grafted onto the surface of IG. This process was carried out in a fluidized bed of IG containing monomers and templates. Figure 1 depicts the basic steps involved in making a MIP. The preparation method for different MIPs is described in the following section.

#### 2.2.1. Phenobarbital MIP

The functional monomer MAA (1.74 mmol, 0.15 mL) and cross-linking monomers MBAA (3.9 mmol, 0.6 g) and EDMA (3.0 mmol, 0.6 g) were dissolved in 1 mL distilled water and 10 mL dichloroethane. To this, 0.1 g of sodium phenobarbital (PB) was added and mixed thoroughly. Finally, 0.25 g IG was added to this solution, kept in a quartz crystal test tube (27 mm ID, Fujirika Kogyo Co., Ltd., Osaka, Japan), and bubbled with nitrogen for 30 min deoxygenation. The quartz tube was placed from the tip of the light guide 2 cm before the UV source for two hours, under continuous stirring and N_2_ bubbling, and saturated with the solvent. After two hours, the template was extracted using a vacuum filtration method. The suspension was cleaned in sequence using (a) 20 mL DMF, (b) 100 mL 1 M NaCl aq. at 60 °C, (c) 100 mL distilled water, and (d) 100 mL methanol. The cleaned MIP was dried using vacuum drying. Non-imprinted polymer (NIP) for PB was created using the same method as MIP, except no template was added.

#### 2.2.2. Levetiracetam MIP

In 10 mL of DMF, the cross-linking monomer MBAA (3.9 mmol, 0.6 g), the functional monomer MAA (1.74 mmol, 0.15 mL), and the functional monomer EDMA (4.0 mmol, 0.8 g) were dissolved. Levetiracetam (LEV) was thoroughly dissolved with 0.1025 g (0.60 mmol). Lastly, 0.24 g of IG was added to this solution, which was then placed in a quartz tube and bubbled with nitrogen for 20 min. Throughout the two hours of continuous stirring and N_2_ bubbling, the quartz tube was positioned 5 cm in front of the UV source. The template was removed using a vacuum filtering technique after two hours. The suspension was cleaned one at a time using 30 mL DMF, 200 mL 1.5 M NaCl at 70 °C, 200 mL distilled water, and 10 mL ethanol. The cleaned MIP was dried using vacuum drying.

#### 2.2.3. Carbamazepine MIP

The cross-linking monomers MBAA (3.9 mmol, 0.6 g), EDMA (4.0 mmol, 0.8 g), and the functional monomer MAA (1.74 mmol, 0.15 mL) were dissolved in 10 mL DMF. This was carefully combined with 0.1184 g (0.501 mmol) of carbamazepine (CBZ). Lastly, 0.25 g of IG was added to this solution and bubbled with nitrogen for 20 min in a quartz tube. The quartz tube was held 5 cm before the UV source for two hours while stirring and bubbling N_2_. A vacuum filtering technique was used to retrieve the template after two hours. The suspension was cleaned in sequence using the following solutions: (a) 30 mL DMF, (b) 200 mL 1.5 M NaCl at 70 °C, (c) 300 mL distilled water, and (d) 10 mL ethanol. Vacuum drying was used to dry the cleaned MIP.

### 2.3. The Disposable Chip Sensor

**Figure 2:**
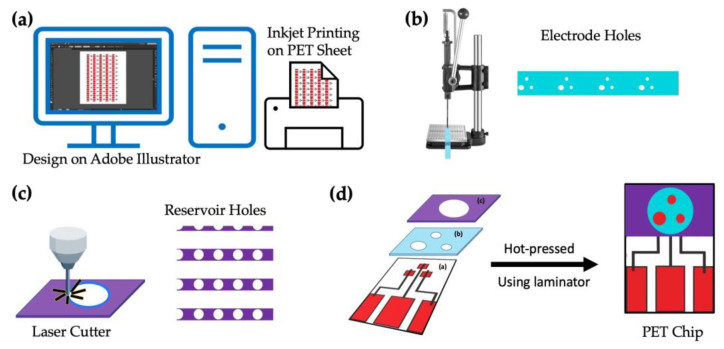
Layout of the PET-Chip design: (**a**) designing the pattern using Adobe illustrator and printing using an inkjet printer, (**b**) punching the electrode holes on the laminate sheet and laminating it on the printed PET-sheet, (**c**) cutting sample reservoir using a laser cutter and laminating the same on the film obtained in (**c**), and (**d**) the final image of the prepared PET chip.

The entire process for fabricating the chip sensor is shown schematically in Figure 2. In the first step, the base-wiring film was prepared by printing silver ink using the inkjet printer on a PET sheet, as seen in Figure 2a. The designs for the base electrodes, electrode holes (reference and counter), and reservoir were all created using Adobe Illustrator CC 2020 (institutional license). The holes for the counter electrodes (2 mm in diameter), reference electrodes (1 mm in diameter), and working electrodes (1 mm in diameter) were then punched using a ToAuto hand-pressed hole punch machine (purchased from Amazon, Japan), as seen in Figure 2b, followed by lamination on the PET sheet printed in Figure 2a. The circular holes for the reservoir (10 mm in diameter) were created by cutting the thermally adhesive PET films using the CO_2_ laser cutter and heat-sealed again over the electrode holes as in Figure 2c. The ready-to-use image of a single chip is seen in Figure 2d.

**Figure 3:**
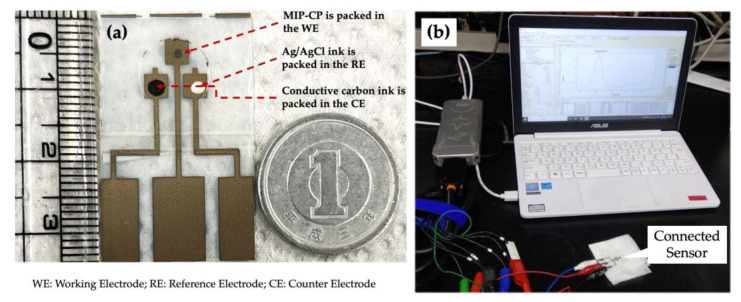
PET-Chip and the sensing setup: (**a**) photo of disposable PET-Chip sensor with size compared to a one Yen coin, and (**b**) DPV setup for the measurement and analysis.

The original picture of the disposable PET chip can be seen in Figure 3. The prepared MIP-CP was packed in the working electrode, as shown in Figure 3a. Every chip was used ‘singly’, i.e., one chip for one concentration. The conductive carbon ink was packed in the counter electrode and dried in an oven at 60° for 1 h to cure it.

### 2.4. Measurement Parameters

Because of its excellent sensitivity, differential pulse voltammetry (DPV) was chosen as the electrochemical method for measuring drug responses in the saline buffer and the blood. Iterative optimization of the electrochemical setup was performed to achieve the best possible outcomes. All the DPVs were performed against silver–silver chloride (Ag/AgCl) reference electrodes, varying the potential from 0.0 V to 0.9 V with a step potential of 10 mV and a scan rate of 20 mV/s. The connection of the chip to the potentiostat is seen in Figure 3b.

Unless otherwise stated, phosphate-buffered saline (pH 7.4) containing 0.1 M NaCl and a 0.05 M mixture of potassium dihydrogen phosphate and disodium hydrogen phosphate were used for the entire experiment to create various concentrations of the analyte under test. The target analyte was first dissolved in 100 μL of physiological saline to prepare the stock solutions for spiked bovine blood samples.

## 3. Results and Discussions

### 3.1. Phenobarbital Sensor

**Figure 4:**
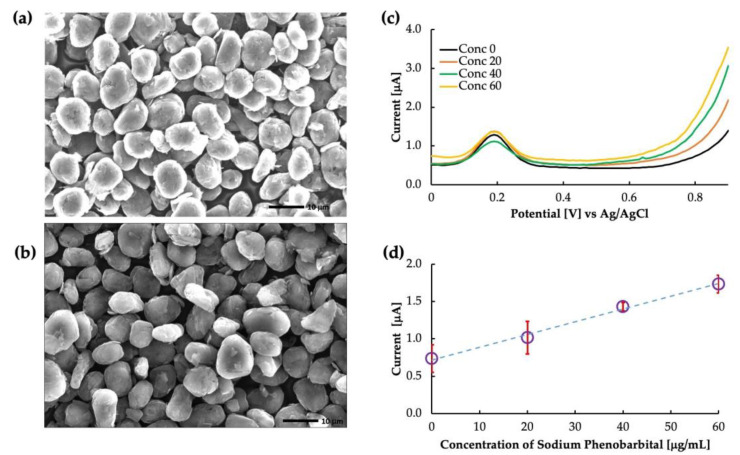
Phenobarbital sensor: (**a**) SEM image of un-grafted carbon, (**b**) SEM image of MIP grafted carbon, (**c**) as-obtained voltammogram, and (**d**) calibration curve drawn by the current obtained at 0.8 V.

Figure 4a,b show the SEM images of the un-grafted carbon and the surface-imprinted molecularly imprinted polymer of phenobarbital grafted onto carbon particles, respectively. The un-grafted particles showed a rough, porous surface with irregularly shaped particles distributed throughout. On the other hand, the grafted particles showed a smoother surface because of the polymer coating. However, the thickness of the MIP layer was too thin to evaluate from the image, and further characterization techniques would be necessary to determine this parameter. Figure 4 depicts the differential voltammograms of PB in the phosphate buffer solution. The peak at approximately 0.2 V is the oxidation peak of ferrocene. The current obtained at a potential of 0.8 V was used to create the calibration curve, as shown in Figure 4d. The calibration showed that the MIP was highly sensitive to PB buffer saline with a coefficient of linearity (*R*^2^) value of 0.99. As the functional monomer, MAA allowed the polymer to interact specifically with the carbonyl group and nitrogen in the phenobarbital molecule. They formed hydrogen bonds with the carboxyl group of MAA, forming a stable complex. This interaction created a specific binding cavity to phenobarbital with high affinity.

**Figure 5:**
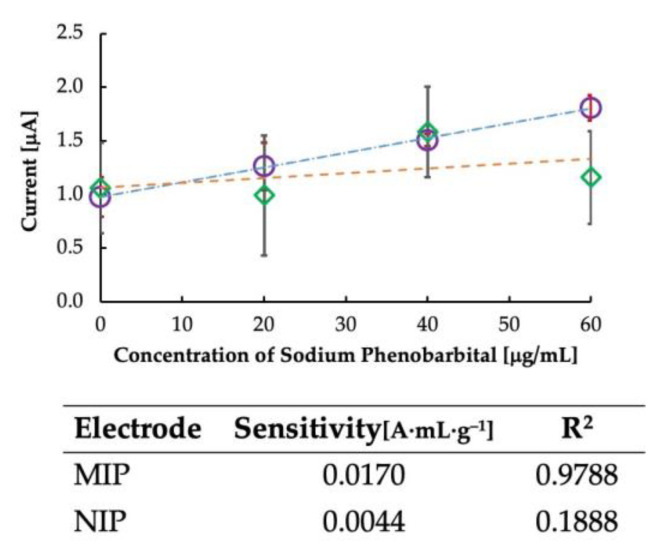
Calibration curve for comparing imprinted and non-imprinted polymer-grafted carbon paste. Circles represent MIP-CP, and rhombuses represent NIP-CP. The table shows the sensitivity and correlation coefficient data of MIP and NIP.

Further, non-imprinted polymers were created as a control experiment to confirm the effect of imprinting, and the results are plotted in Figure 5. It is clear from the figure that the sensitivity of the sensor was dependent on the imprinting of the MIP. Since NIPs have no imprinting sites, the sensitivity was not dependent on the rebinding of the template to the MIP cavities, and some random current was generated. Thus, the imprinted polymer is vital in determining the sensor’s sensitivity. The sensitivity of the MIP-CP and the NIP-CP is shown in Figure 5. It is clear from the table that the sensitivity of MIP-CP was higher than that of NIP-CP, with a linearity coefficient of >0.97.

**Figure 6:**
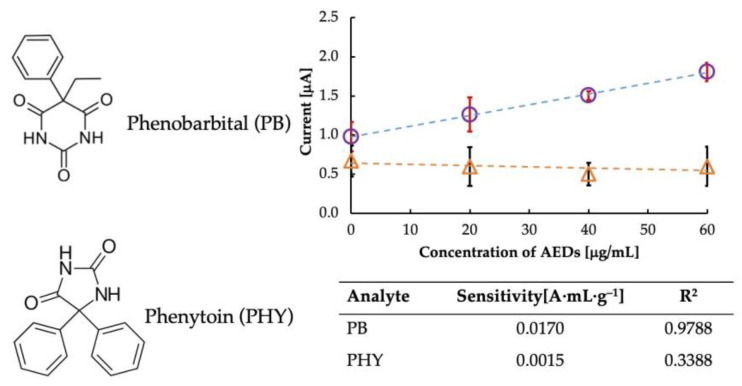
Comparison of selectivity of the sensor towards Phenobarbital (circles) and its analog, Phenytoin (triangles).

Selectivity is an important parameter when deciding on an ideal sensor. Therefore, we performed a selectivity test of the sensor in the presence of phenytoin (PHY), a structurally similar drug, as shown in Figure 6. Since phenobarbital interacts with imprinted cavities in MIP-CPs, the results revealed that MIP-CPs were material specific concerning phenobarbital but not so concerning other materials, as evident from the sensitivity data listed in the table in Figure 6. PHY has a diphenylhydantoin structure which is different from that of PB. Since the imprinted cavity was designed explicitly for PB, upon the interaction of the MIP cavities with PHY, there was no specific electrochemical change in the MIP. Thus, the MIP in the PB sensor did not recognize the molecular structure of PHY and hence did not exhibit selectivity toward it.

**Figure 7:**
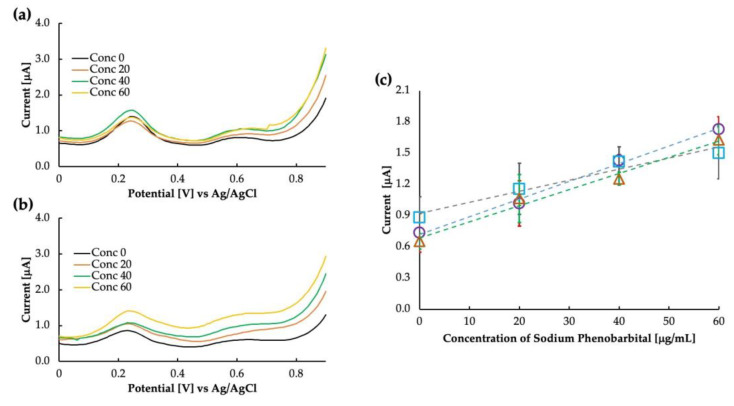
Differential pulse voltammograms as obtained in (**a**) bovine whole blood and (**b**) bovine plasma, and (**c**) calibration curve comparing the sensitivity in buffer saline (circles), bovine whole blood (squares), and bovine plasma (triangles).

It is essential to extensively understand the sensor’s selectivity when targeting the use of the sensor in whole blood, as the various components of blood and other contaminants of the blood may interact with the sensor’s sensitivity. We tested the sensor in whole bovine blood and bovine plasma to verify whether our sensor works in such a complex matrix. The result of this is shown in Figure 7. Compared with bovine whole blood and bovine plasma, the response of the MIP was almost similar. For bovine whole blood, the intercept was higher at 0.92 μA compared with buffer saline and bovine plasma, which had intercept values of 0.71 μA and 0.68 μA, respectively. As the intercept value increased, the linearity was also reduced to 0.96 for whole bovine blood. The exact values for sensitivity and linearity are added in Table 1. It is possible that some of the components of blood, such as uric acid (although not so high in amount in bovine whole blood) and some lipophilic contents of the blood interact with the sensitivity. Further clarification about the effect of blood components can be obtained by observing the sensitivity of the sensor in bovine serum, which is currently out of the scope of this paper.

### 3.2. Levetiracetam and Carbamazepine Sensors

**Figure 8:**
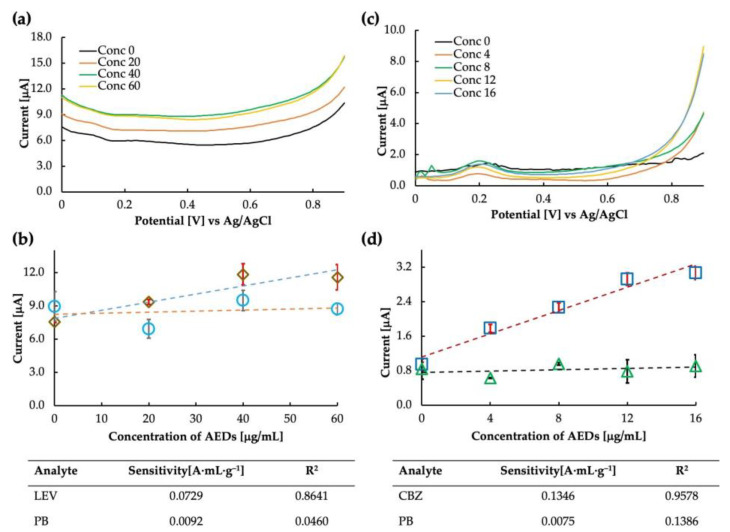
Levetiracetam sensitivity analysis: (**a**) differential pulse voltammogram of the MIP-CP sensor for Levetiracetam obtained against Ag/AgCl, and (**b**) calibration curve obtained at 0.8 V in levetiracetam (rhombuses) and phenobarbital (circles). Carbamazepine sensitivity analysis: (**c**) differential pulse voltammogram of the MIP-CP sensor for carbamazepine obtained against Ag/AgCl, and (**d**) calibration curve obtained at 0.8 V in carbamazepine (squares) and phenobarbital (triangles).

The prepared levetiracetam MIP was used as the electrode for the sensor chip. The voltammogram and the calibration curve obtained in buffer saline are shown in Figure 8a,b. It is clear from the calibration curve (drawn at 0.8 V) that the MIP showed some sensitivity to LEV; however, the sensitivity at the higher concentration value of 60 μg/mL seemed to drop suddenly. The MIP cavities may have become saturated at such a high concentration, and no further increase in current could be observed. Another possibility is to improve the polymer matrix’s flexibility by improving the crosslinking monomer selection so that more MIP can rebind at higher concentrations and a sensitive current can be obtained. The LEV sensor’s calculated sensitivity and correlation coefficient were 0.0729 A·mL·g^−1^ and 0.8461, respectively. The binding between the analyte molecule and the MIP cavities results from various chemical interactions, such as hydrogen bonding, electrostatic interactions, and van der Waals forces. When rebinding, it is possible that the specific functional groups in the LEV molecule, such as amine and carbonyl, form hydrogen bonds with the complementary functional groups in the MIP cavities.

The LEV-MIP was tested for selectivity against phenobarbital, and it was found that the response of the sensor in PB was unstable, accounting for the excellent selectivity of the designed MIP. Thus, the MIP sensor for LEV can be implemented for further analysis. Additionally, we observed that the same DPV parameters may only work for some types of MIP sensors [21]. Therefore, parameter optimization may also be a crucial step in finding the sensitivity of the sensor. Parameters such as scan rate, step potential, and accumulation time often affect the response of the sensor. All the parameters must be optimized to obtain the best result. Since this is a preliminary report on the feasibility of the LEV sensor, we expect to improve the parameters over time (in addition to optimizing the MIP).

MIP-carbon paste sensors for carbamazepine were tested for sensitivity. We tried the sensor only once and found it highly sensitive to CBZ. The current was measured, and the intensity at 0.8 V was plotted on the calibration curve. The calculated sensitivity and correlation coefficient of the CBZ sensor were 0.1346 A·mL·g^−1^ and 0.9578, respectively. The selectivity test of carbamazepine and phenobarbital showed that the MIP was highly selective towards CBZ. Both the sensitivity and selectivity plots can be seen in Figure 8c,d.

Interestingly, the sensitivity trends of LEV and CBZ at higher concentrations were similar and tended to be lower than levetiracetam MIP obtained at low concentrations. One possible explanation for the high sensitivity and selectivity of the CBZ MIP sensor is the specific interaction between the functional monomer and the CBZ molecule. The functional monomer used in the synthesis of the MIP was methacrylic acid (MAA), which has a carboxylic acid group and is thought to be hydrogen bonded to the nitrogen of the seven-membered ring of CBZ (MAA is unlikely to bond strongly to the amide group of CBZ in DMF, which is also an amide compound), leading to the formation of selective recognition sites (cavities) within the polymer matrix. Further, the lack of sensitivity of CBZ and LEV MIPs to phenobarbital could be due to the differences in the chemical structure and binding modes of PB compared with the target molecules, or the suboptimal cavity size and shape of the MIPs for PB binding.

It should be noted that for sample preparation, we used 1 mL DMF to dissolve 10 mg of CBZ and then dissolved it in 200 mL buffer saline to dilute it and make several smaller concentration samples. Although the amount of DMF is so little and diluted, there may be a possibility that the polymer matrix of the MIP is affected by the presence of DMF in the measurement solution, which may thus affect the sensitivity. The best method to dissolve CBZ needs to be established. Further, optimizing the MIP composition and measurement parameters is essential for a sensitive MIP. We plan to improve the MIP and the measurement parameters to obtain a stable and sensitive CBZ sensor. Table 2 shows a summary of the comparison of our sensor with some of the available reports on AED sensors. However, because of limited work on the Levetiracetam MIP sensor, sufficient information could not be gathered.

The outcomes of the tests performed on our MIP-based AED sensors have proven entirely satisfying. The sensor quickly determines the concentration without reagents, thus reducing the complexities of adding a biological agent for detection. We used ferrocene in silicone oil as the redox marker in our experiment; the details can be found in our previous work [33]. When ferrocene is mixed with silicon oil, the large surface area of the oil allows many ferrocene molecules to be incorporated into the system, resulting in increased sensitivity to changes in the local electrochemical environment. The ferrocene molecules can participate in redox reactions with the surrounding environment, leading to changes in the oxidation state of the iron complex. These changes can be detected as changes in the electrochemical signal, which can be used as a marker for the presence of redox-active species in the surrounding environment. In the presence of an analyte drug, the redox reactions of the redox marker are affected by the interactions between the analyte drug and the MIP-CP. The imprinted cavities in the MIP-CP can bind to the analyte drug, leading to changes in the local electrochemical environment. As the concentration of the analyte drug increases, the interactions between the analyte drug and the imprinted cavities in the MIP also increase. This leads to an increase in the amount of analyte drug bound to the MIP, which results in a more significant change in the local electrochemical environment and a more considerable change in the DPV current and vice-versa.

The PB sensor has a high selectivity in addition to having a high sensitivity. In most instances, the degree of sensitivity exhibited by MIPs is determined by the monomers chosen for polymerization. However, it can be attributed to several factors, including the imprinted cavities’ specificity, the imprinted polymer’s large surface area, and its high binding affinity for the target molecule. The specificity of the imprinted cavities is achieved through the precise control of the monomer composition and polymerization conditions, which dictate the size and shape of the imprinted cavities. The large surface area of the imprinted polymer provides many recognition sites for the target molecule, increasing the likelihood of binding and leading to a higher signal. Additionally, the high binding affinity of the imprinted cavities is due to the formation of hydrogen bonds and van der Waals forces between the target molecule and the imprinted cavities.

Nonetheless, when measured in an aqueous media, the sensitivity is hindered by the high polarity of water because water tends to weaken the hydrogen bonds between the MIP and the template. This could be one of the reasons for observing saturated or lower responses of levetiracetam and carbamazepine at higher concentrations. Optimizing the monomers (ratio and choice of the monomers), polymerization time, porogen selection, etc., can improve the MIP-CP. An expanded study on selectivity, NIP-CP, and results in whole bovine blood or rat plasma will be communicated soon.

**Table 2:** Comparison of some of the AEDs in this work.

Target Drug	Electrode Material	Detection Method	Linear Range (mol·L^−1^)	Preparation Method	Reagent Required	Operation Time (min)	Plasma/Serum Separation	Refs.
PB	Nickel-modified glassy carbon	DPV	1.4 × 10^−7^–1.3 × 10^−4^	Thermal polymerization	Yes	>8	NA	[34]
	Glassy carbon	DPV	1.0 × 10^−8^–1.8 × 10^−4^	Electropolymerization	Yes	NA	NA	[35]
	Carbon paste	HPLC	8.6 × 10^−5^–4.3 × 10^−4^	-	Yes	NA	Yes	[36]
	Carbon paste	DPV	0–3.0 × 10^−4^	Photopolymerization	No	2	No	This work
CBZ	Glassy carbon	CV, SWV	NA	Electropolymerization	Yes	NA	NA	[20]
	Silver nanosphere on glassy carbon	CV, DPV	1.0 × 10^−9^–5.0 × 10^−6^	Electropolymerization	NA	NA	Yes	[37]
	Carbon paste	DPV	0–5.0 × 10^−5^	Photopolymerization	No	2	No	This work

CV: cyclic voltammetry, DPV: differential pulse voltammetry, SWV: square wave voltammetry, NA: not available.

## Figures and Tables

**Table 1:** Comparison of phenobarbital sensing in various matrixes.

Matrix	Sensitivity [A·mL·g^−1^]	R^2^
Buffer saline	0.0170	0.9788
Bovine whole blood	0.0106	0.9560
Bovine plasma	0.0155	0.9826

## Data Availability

Not Applicable.
